# HTLV-1 proviral integration sites differ between asymptomatic carriers and patients with HAM/TSP

**DOI:** 10.1186/1743-422X-11-172

**Published:** 2014-09-30

**Authors:** Heather A Niederer, Daniel J Laydon, Anat Melamed, Marjet Elemans, Becca Asquith, Masao Matsuoka, Charles RM Bangham

**Affiliations:** Department of Immunology, Wright-Fleming Institute, Imperial College London, London, W2 1PG UK; Institute for Viral Research, Kyoto University, Kyoto, 606-8507 Japan

**Keywords:** HTLV-1, Human T cell lymphotropic virus-type 1, HBZ, HTLV-1 basic leucine zipper factor, HAM/TSP, HTLV-1-associated myelopathy/tropical spastic paraparesis, Integration site, CD8+ T cell

## Abstract

**Background:**

HTLV-1 causes proliferation of clonal populations of infected T cells in vivo, each clone defined by a unique proviral integration site in the host genome. The proviral load is strongly correlated with odds of the inflammatory disease HTLV-1-associated myelopathy/tropical spastic paraparesis (HAM/TSP). There is evidence that asymptomatic HTLV-1 carriers (ACs) have a more effective CD8 + T cell response, including a higher frequency of HLA class I alleles able to present peptides from a regulatory protein of HTLV-1, HBZ. We have previously shown that specific features of the host genome flanking the proviral integration site favour clone survival and spontaneous expression of the viral transactivator protein Tax in naturally infected PBMCs ex vivo. However, the previous studies were not designed or powered to detect differences in integration site characteristics between ACs and HAM/TSP patients. Here, we tested the hypothesis that the genomic environment of the provirus differs systematically between ACs and HAM/TSP patients, and between individuals with strong or weak HBZ presentation.

**Methods:**

We used our recently described high-throughput protocol to map and quantify integration sites in 95 HAM/TSP patients and 68 ACs from Kagoshima, Japan, and 75 ACs from Kumamoto, Japan. Individuals with 2 or more HLA class I alleles predicted to bind HBZ peptides were classified ‘strong’ HBZ binders; the remainder were classified ‘weak binders’.

**Results:**

The abundance of HTLV-1-infected T cell clones in vivo was correlated with proviral integration in genes and in areas with epigenetic marks associated with active regulatory elements. In clones of equivalent abundance, integration sites in genes and active regions were significantly more frequent in ACs than patients with HAM/TSP, irrespective of HBZ binding and proviral load. Integration sites in genes were also more frequent in strong HBZ binders than weak HBZ binders.

**Conclusion:**

Clonal abundance is correlated with integration in a transcriptionally active genomic region, and these regions may promote cell proliferation. A clone that reaches a given abundance in vivo is more likely to be integrated in a transcriptionally active region in individuals with a more effective anti-HTLV-1 immune response, such those who can present HBZ peptides or those who remain asymptomatic.

**Electronic supplementary material:**

The online version of this article (doi:10.1186/1743-422X-11-172) contains supplementary material, which is available to authorized users.

## Background

Human T cell lymphotropic virus-type 1 (HTLV-1) is estimated to infect over 10 million people [1], and is endemic in sub-Saharan Africa, the south islands of Japan, the Caribbean and parts of South America. HTLV-1 is primarily found in CD4^+^ T cells, where predominantly only a single copy of the virus integrates into the genome [2]. The virus is almost 100% cell-associated, and the viral burden is defined as the fraction of PBMCs that carry the integrated provirus, termed the proviral load (PVL). Infected cells proliferate in vivo, producing clonal populations of cells, each defined by its unique proviral integration site. The viral regulatory proteins Tax and HTLV-1 basic leucine zipper factor (HBZ) are known to drive proliferation of the infected cells [3–5].

More than 90% of HTLV-1-infected individuals remain lifelong asymptomatic carriers (AC), but 1–6% develop an aggressive malignancy known as adult T cell leukaemia/lymphoma (ATLL). A further 0.25 to 4% develop a chronic inflammatory disease of the central nervous system, HAM/TSP, characterised by a slowly progressive spastic paraparesis with pain and neurogenic bladder disturbance [6]. Risk factors for HAM/TSP include female gender and high PVL [7].

There is strong evidence that the CD8^+^ T cell response is important in limiting PVL, and reducing the risk of HAM/TSP [8], although innate immunity also plays a role in the host response to HTLV-1 [9]. Certain HLA class I alleles are associated with a reduction in PVL and prevalence of HAM/TSP, in particular HLA-A∗02 and Cw∗08 in a population from Southern Japan [10]. Tax is the dominant CD8^+^ T cell target antigen of HTLV-1 [11, 12]: Tax escape mutations in the HLA-A2-restricted epitope Tax 11-19 are more frequent in individuals with the HLA-A2 allele [13], and Tax expression is frequently silenced in the expanded clone in ATLL by mutations in *tax* or methylation or deletion of the 5’LTR [14–17]. The rate of lysis of Tax^+^CD4^+^ cells by CD8^+^ cells has been inversely correlated with PVL [18], although Tax mRNA is virtually undetectable directly ex vivo. Individuals who remain asymptomatic were shown to have a lower PVL than those with HAM/TSP at a given lysis rate [18], and had a greater CD8^+^ T-cell lytic efficiency as measured by proportion of Tax-specific CTL which degranulate when exposed to their cognate epitope ex vivo [19].

Unlike Tax, HBZ expression is uniformly maintained in HTLV-1-infected T cells, including ATLL cells [4], and this expression correlates with PVL in both ACs and patients with HAM/TSP [20]. On average, HBZ peptides bind to HLA class I alleles with lower affinity than Tax peptides, and the frequency of HBZ-specific CD8+ T cells [21] is correspondingly lower. HBZ expression may be maintained because it can drive expansion of an infected clone without presenting a strong target to the CD8^+^ T cell response. The frequency of HLA class I alleles that are predicted to strongly bind HBZ peptides is greater in ACs than patients with HAM/TSP, and is inversely correlated with PVL in each group [21]. These observations suggest that a CD8^+^ T-cell response to the HBZ protein is protective against HTLV-1-associated inflammatory disease.

The equilibrium abundance in vivo of a particular HTLV-1-infected T-cell clone is the result of the interplay between the proliferation of the clone and counter-selection by the host response, chiefly the CD8^+^ T cell response. Both factors are governed by the program of proviral expression by the clone. Since the proviral sequence is very stable [22], the chief unique attribute of each HTLV-1-infected T-cell clone is the genomic position of the integrated provirus – the proviral integration site. Specific features of the genomic environment of the HTLV-1 proviral integration site are associated with the frequency of spontaneous reactivation of Tax expression ex vivo [23]. Integration in the same transcriptional orientation as a flanking host gene is associated with suppression of Tax expression: same-sense orientation is more frequent in high-abundance clones, and more frequent in vivo than during in vitro infection, suggesting that this orientation confers a selective advantage by allowing escape from the Tax-specific CD8^+^ T cell response [23]. There are no published data on the influence of the integration site environment on HBZ expression.

Since the genomic integration site influences proviral expression, we reasoned that the selection pressure exerted by a protective immune response will alter the abundance of clones which have integration site genomic environments with certain characteristics. Our previous reports were neither designed nor powered to examine the relationship between the integration site environment and either disease status or host immunogenetics. In this study, we investigated the differences in integration site environment between Japanese individuals who remained AC and those who developed HAM/TSP, and between those that differed in their capacity to present HBZ peptides on protective HLA class I alleles. We report that integration sites in genes and active regions are significantly more frequent in ACs than in patients with HAM/TSP, even after accounting for clone abundance and PVL. Integration sites in genes are also more frequent in strong HBZ binders.

## Results

### Possession of HBZ-binding HLA class I alleles was associated with reduced HTLV-1 proviral load, but did not affect oligoclonality of the HTLV-1 infected cell population or total number of clones by load

We have previously shown that in both ACs and patients with HAM/TSP in Kagoshima prefecture (Southern Japan), the number of HLA class I A and B alleles predicted to bind HBZ epitopes is inversely correlated with PVL [21]. Here, we extended this analysis with a second southern Japanese AC cohort, from nearby Kumamoto prefecture (Table 1). The predicted ability of individual HLA class I A and B alleles to bind HBZ epitopes was determined using the rank of the top HBZ-binding peptide in the peptide binding prediction software Metaserver, as previously described [21] (Table 2). We then used linear regression to analyse the relationship between the number of HBZ-binding alleles and PVL. In the Kumamoto cohort, as in the two Kagoshima cohorts, there was an inverse correlation between log PVL and the number of HBZ-binding alleles (linear regression: slope = -0.12, Table 3). This correlation was significant in a regression analysis of the combined Kagoshima cohorts (p = 0.02) as previously reported, and all three cohorts combined (p = 0.006), although it did not reach significance in the smaller Kumamoto cohort alone.

**Table 1:** **Total patients analysed and integration sites identified**

	Kagoshima						Kumamoto		
	HAM/TSP			AC			AC		
**Number of patients in:**	Strong HBZ	Weak HBZ	***Total***	Strong HBZ	Weak HBZ	***Total***	Strong HBZ	Weak HBZ	***Total***
Whole cohort	81	148	***229***	92	110	***202***	34	64	***98***
All LM-PCR	42	62	***104***	41	59	***100***	34	64	***98***
LM-PCR QC pass	39	56	***95***	30	38	***68***	24	51	***75***
Number of UIS* with abundance:									
<1 per 10,000 PBMC	7954	12341	***20295***	1451	5485	***6936***	4572	9744	***14316***
1-10 per 10,000 PBMC	10051	12429	***22480***	2178	2515	***4693***	1034	1853	***2887***
>10 per 10,000 PBMC	219	364	***583***	102	67	***169***	42	40	***82***
All			***43358***			***11798***			***17285***

*UIS identified from LM-PCR QC pass samples only.

AC: Asymptomatic carrier, UIS: Unique integration site, QC pass: samples that passed LM-PCR quality controls (QC).

**Table 2:** **HLA class I alleles divided by HBZ epitope binding status**

Strong HBZ binding alleles	Weak HBZ binding alleles
A0201, A0206, A0207, A0210	A0101
A2601, A2602, A2603	A0203
A3001	A0301, A0302
A3303	A1101
	A2402
	A3101
	A3201
B3701	B0702
B4001, B4002, B4006	B1301, B1302
B41	B1501, B1507, B1511, B1518
B4403	B2704
	B3501, B3520, B3532/B3568
	B3802
	B3901
	B4003, B4009/B4027/B40105
	B4601
	B4801
	B5101
	B5201
	B5401
	B5502, B5504
	B5601, B5605
	B5801
	B5901, B5902
	B6701

**Table 3:** **Linear regression of HBZ binding alleles to PVL**

Outcome	Predictor	Cohort*	n	Slope	p value	Controlling for:
Log PVL	# HBZ binding alleles	Kagoshima HAM, AC	HAM: 221 AC: 200	-0.12	0.021	Age, sex, disease status
Log PVL	# HBZ binding alleles	Kumamoto AC	AC: 98	-0.12	0.28	
Log PVL	# HBZ binding alleles	Kagoshima HAM, AC & Kumamoto AC	HAM: 229 AC: 300	-0.13	0.0058	Disease status

*Only individuals who had all the relevant information were included in the regression. Age, sex and disease status were included as factors where possible as they may vary with PVL.

Age and sex information was not available for the Kumamoto AC cohort.

PVL: proviral load, AC: Asymptomatic carrier, #: number of.

We used our recently developed high-throughput protocol [24] to map and quantify the abundance of unique proviral integration sites in the HAM/TSP cohort and the two AC cohorts (Table 1). Sufficient DNA was not available from all subjects, but the median PVL of the HAM/TSP samples that passed LM-PCR quality controls (QC) was not significantly different from that of the full HAM/TSP cohort (Additional file 1: Figure S1A). In the AC cohorts the median PVL of successfully analysed samples exceeded that of all ACs, because a proportion of ACs have a PVL too low to permit accurate quantification [7] and LM-PCR. Since this reduces the difference in median PVL between the analysed AC and HAM/TSP cohorts, our results represent a conservative estimate of the difference between ACs and patients with HAM/TSP.

**Figure 1:**
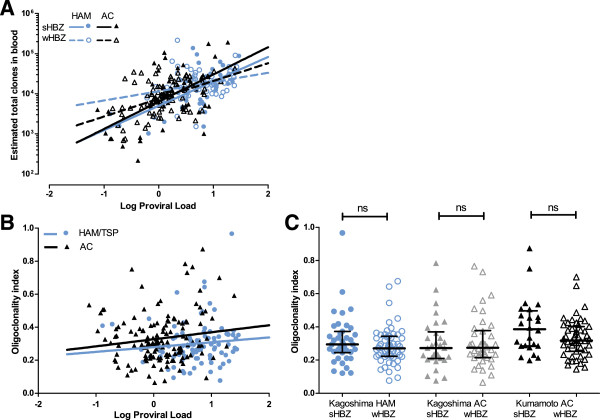
**Host disease status and HBZ-binding capacity do not alter total unique HTLV-1-infected clones or oligoclonality.** HTLV-1 infected Japanese asymptomatic carriers (AC; triangles) from Kagoshima and Kumamoto prefectures and HAM/TSP patients (circles) from Kagoshima were stratified on the basis of predicted HBZ peptide binding affinity of host HLA class I alleles (strong binders, sHBZ, filled symbols; weak binders, wHBZ, open symbols). Each symbol represents one individual. Genomic DNA samples were processed by sonication-based LM-PCR and samples passing data quality checks were analysed further, as previously described ( [23, 24]). **(A)** The DivE method was used to estimate the total number of clones in the blood in each subject; the total clone number was positively correlated (Spearman correlation) with proviral load in each cohort both HAM (blue, p = 0.0004) and AC (black, p = 0.0001)). There was no significant difference in slope of the linear regression line between AC and HAM/TSP cohorts (likelihood ratio test, p = 0.30) or between sHBZ (continuous line) and wHBZ (dashed line) in the combined AC and HAM/TSP cohorts (likelihood ratio test, p = 0.06) indicating that there is no systematic difference in total clone number by disease status or HBZ binding status. **(B)** The oligoclonality index (OCI), which quantifies the diversity in observed clone abundance in each sample, was not significantly correlated with proviral load in ACs or HAM/TSP (Spearman correlation) and there was no significant difference in slope of the linear regression line between AC and HAM/TSP cohorts. **(C)** The median OCI did not differ significantly by HBZ binding status within individual cohorts (Mann-Whitney U test). Bars denote median +/- interquartile range.

Since strong HBZ peptide binding is associated with efficient control of PVL, we tested whether this also altered the frequency distribution of the infected cell population. Individuals were designated strong HBZ binders if they carried two or more predicted HBZ binding HLA class I alleles. The total number of HTLV-1 proviruses mapped in strong HBZ binders was similar to that in weak HBZ binders in each cohort (Additional file 1: Figure S1B), allowing unbiased estimates of clone structure. We estimated the total number of integration site clones in the blood using our novel in silico approach [25]. The estimated number of clones correlated with PVL in all cohorts (Figure 1A). There was no significant difference in estimated total number of clones in the blood, after stratifying by PVL, between AC and HAM/TSP cohorts, or between strong HBZ binders and weak HBZ binders (Figure 1A). The Oligoclonality Index (OCI) quantifies the diversity in clone abundance in an infected T cell population: an OCI approaching 1 indicates an essentially monoclonal population, whereas an OCI of 0 indicates that all clones have the same abundance. Consistent with our previous observations [24], OCI did not correlate with PVL and did not differ between ACs and HAM/TSP cohorts (Figure 1B); there was also no difference in OCI between strong and weak binders of HBZ in any cohort (Figure 1C).

### Integration in transcriptionally active areas was positively selected in asymptomatic carriers and was associated with high clonal abundance

**Figure 2:**
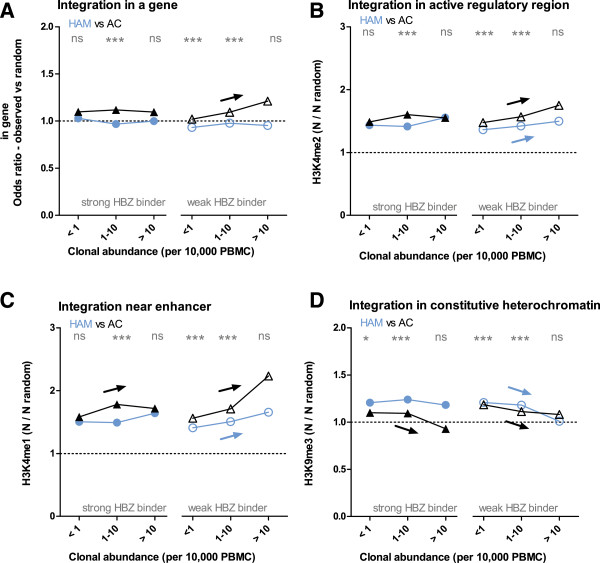
**Transcriptionally active integration sites associate with AC status and clone abundance in weak HBZ binders.** HTLV-1 unique integration sites from Japanese asymptomatic carriers (AC, black triangles) from Kagoshima and Kumamoto were compared to those from HAM/TSP patients (blue circles, from Kagoshima). Integration sites were stratified on the basis of predicted HBZ peptide binding affinity of host HLA class I alleles (strong binders, sHBZ, filled symbols; weak binders, wHBZ, open symbols) and binned by absolute abundance. Data is expressed versus an in silico generated random integration site dataset. **(A)** AC individuals (Kagoshima and Kumamoto cohorts combined) had a greater proportion of integration sites in genes than HAM/TSP patients (chi-squared test). Percentage of clones with integration sites in genes was correlated with clone abundance only in individuals with HLA class I alleles which could not bind HBZ (arrow, significant chi-squared test for trend). **(B)** Asymptomatic carriers had a higher frequency than HAM/TSP of H3K4me2 marks, enriched in transcriptionally active areas, within 10 Kb of integration sites (Mann-Whitney U test), and **(C)** a higher frequency of H3K4me1 marks, associated with enhancers. Mean epigenetic mark frequency near integration sites in a bin is divided by frequency near random sites. **(D)** In contrast, AC had a lower frequency of H3K9me3 marks, associated with constitutively heterochromatic DNA. Spearman correlation shows a significant (arrow) association between epigenetic mark frequency and log absolute abundance. Statistical comparisons AC vs HAM by Mann-Whitney U test after correction for multiple testing: * 0.05 > p > 0.01, ** 0.01 > p > 0.001, *** p < 0.001.

Detected integration sites were binned according to their absolute abundance in 10,000 PBMCs (Additional file 1: Figure S1C). In this study, we compared the frequency of transcriptionally active sites between ACs and HAM/TSP patients, after stratifying by host HBZ binding strength and absolute clonal abundance. There was no significant difference in the genomic environment of integration sites between the AC cohorts from Kumamoto and Kagoshima (Additional file 1: Figure S2B, Additional file 1: Figure S3A), which enabled combination of the two cohorts for further statistical analysis. We previously reported that the proportion of integration sites located within a gene and in active genomic regions increased in clones which had a higher absolute abundance; conversely that high abundance clones had a reduced frequency of sites modified with inhibitory epigenetic marks [24]. Here, we also observe significant correlations between clonal abundance and integration in a gene and frequency of epigenetic marks associated with active genomic regions (Figure 2, Additional file 1: Figure S3).

The proportion of integration sites within genes was significantly higher in ACs than in HAM/TSP patients (Figure 2A). The proportion of integration sites within genes did not correlate with PVL (Additional file 1: Figure S2A), suggesting that the difference between ACs and patients with HAM/TSP could not be explained simply by a lower PVL in ACs. There was no significant difference between ACs and patients with HAM/TSP in the proportion of integration sites in the same transcriptional orientation as the nearest host gene (Additional file 1: Figure S2C) or nearby transcriptional start site (Additional file 1: Figure S2D).

Compared to patients with HAM/TSP, integration sites from ACs had higher counts of H3K4me2 and H3K27ac marks within 10 Kb (indicating active regulatory regions and open chromatin; Figure 2B, Additional file 1: Figure S3B) and higher counts of H3K4me1 (associated with enhancers; Figure 2C). Conversely, integration sites had higher counts of H3K9me3 (associated with constitutively repressed chromatin) in patients with HAM/TSP than ACs (Figure 2D). There was no difference in the frequency of H3K27me3 marks, which are associated with facultative heterochomatin (Additional file 1: Figure S3C). Within genes, integration sites marked by H4K20me1 (enriched at 5′ ends of actively expressed gene bodies) were more frequent in ACs (Additional file 1: Figure S3D), but the percentage of integration sites marked by H3K36me3 (enriched at 3′ ends of actively expressed gene bodies) did not consistently correlate with either clone abundance or disease status (Additional file 1: Figure S3E). The increased frequency of active sites and sites in genes in AC individuals was also significant when the characteristics of all integration sites within a patient were averaged, and the cohorts analysed at the patient level (Additional file 1: Figure S4).

Since transcriptional activity of the genome is correlated with gene density, we used a multivariable logistic regression model to simultaneously test the independence of association of integration in a gene, active region and heterochomatin with disease status. This association was tested both at the level of individual integration sites, and using a single value per patient representing the averaged integration sites from that patient. The results showed that integration within a host gene and within active and inhibitory genomic regions were each independently associated with disease status, after controlling for clone abundance and host PVL and predicted HBZ binding affinity (Table 4 and Additional file 1: Figure S3F). Controlling for HBZ affinity using counts of HBZ binding alleles per patient, rather than a strong/weak designation, gave a very similar significance and odds ratio.

**Table 4:** **Proviral integration within genes and active genomic regions are independently associated with disease status**

Data	n	Outcome		In gene*	# H3K4me2	# H3K9me3
Integration sites	AC: 29083 HAM/TSP: 43358	AC vs HAM	OR	1.08	1.13	0.88
			p value	4.2×10^-5^	4.8×10^-7^	2.1×10^-6^
Patients	AC: 143 HAM/TSP: 95	AC vs HAM	OR	1.12	1.14	1.0
			p value	0.010	0.011	1.0

Additional factors controlled in multiple logistic regression model: Host HBZ binding status and proviral load, and log absolute clone abundance (per integration site) or log average absolute clone abundance (per patient).

*Percent of integration sites in gene per patient in patient-level analysis.

#: Number of specified epigenetic marks within 10 Kb of integration site. Averaged per patient in patient-level analysis.

OR: Odds ratio, AC: Asymptomatic carrier.

We next compared the frequency of transcriptionally active sites between individuals with strong or weak HBZ binding potential. Integration sites from HBZ strong binders were more likely to be in a gene than those from weak binders, even when host disease status and clone absolute abundance were taken into account (Table 5); there was no significant association with frequency of H3K4me2 or H3K9me3 epigenetic marks. There was a similar odds ratio between strong and weak HBZ binders in the percentage of integration sites in a gene per patient, although at this level of individual patients the association did not reach statistical significance.

**Table 5:** **Proviral integration within genes and active genomic regions is associated with disease status**

Data	n	Outcome		In gene*
Integration sites	sHBZ: 27603 wHBZ: 45439	sHBZ vs wHBZ	OR	1.04
			p value	0.007
Patients	sHBZ: 93 wHBZ: 146	sHBZ vs wHBZ	OR	1.03
			p value	0.2

Additional factors controlled in multiple logistic regression model: Disease status and log absolute clone abundance (per integration site) or log average absolute clone abundance (per patient).

*Percent of integration sites in gene per patient in patient-level analysis.

OR: Odds ratio, sHBZ: strong HBZ binder, wHBZ: weak HBZ binder.

## Discussion

At all levels of clone abundance, ACs had a significantly higher frequency than patients with HAM/TSP of integration sites within host genes and in genomic regions marked by activating epigenetic modifications. This enrichment was associated with disease status per se and was independent of variation in proviral load. The odds ratio of this enrichment in each case was modest (~1.1), however the finding that both integration in a gene and integration in an active genomic region were independently associated with AC status strongly suggests a consistent underlying biological mechanism. The question arises: what are the forces that favour selective survival of these integration sites (i.e. in active transcribed regions) in ACs?

We previously reported that Tax-silenced proviruses were more likely to lie in the same orientation as a flanking host gene or nearby upstream transcriptional start site; we concluded that this effect may be attributed to transcriptional interference [23]. The integration site locations that inhibit Tax expression were also associated with increased clone abundance. Consistent with these two findings, more abundant clones were less likely to express Tax [23]. These observations raised the possibility that a provirus integrated in the same orientation as a host gene might enjoy a selective advantage in individuals with an effective immune response because it is less exposed to the strong anti-Tax CD8^+^ T cell response. However, the integration site environments associated with Tax silencing were not those associated with an increased frequency in ACs compared to HAM/TSP in this study.

A second possibility is that a more efficient cellular and innate immune response in ACs [8] means that a clone needs a greater proliferative capacity to reach a given absolute abundance. Interestingly, we also observe an increase in the percentage of integration sites in a gene amongst integration sites from individuals with strong predicted HBZ binding capacity compared those less likely to bind HBZ epitopes, particularly in the less abundant clones. Increasing numbers of HLA class I alleles able to present HBZ are associated with decreasing PVL, suggesting that there is greater control of infected cells in these patients [21]. Integration sites located in genes and active genomic regions are associated with increased clone abundance ( [24], and this study): we postulate that these environments support virus-driven cell proliferation allowing clones to survive under stronger immune control.

Tax is known to drive proliferation of the infected cell; could the clones which selectively survive in ACs express more Tax? We previously observed, in a very small sample of integration sites (n = 40, [26]), that Tax-expressing cells had a higher frequency of integration sites in genes. However, with the advent of high-throughput sequencing, we have recently shown that clones that spontaneously express Tax ex vivo have a minor increase in the frequency of integration in a gene and in regions with activating epigenetic marks ( [23] and AM, unpublished observations). Since we have previously observed that more abundant clones are less likely to express Tax [23], Tax expression is unlikely to completely account for the success of these integration site clones. HBZ is also known to drive cell proliferation [4], and integration in a gene or active region may also promote increased expression of HBZ. To definitively determine, however, whether transcriptionally active regions promote increased HBZ expression will require high-throughput sorting and integration site analysis of HBZ expressing clones directly ex vivo: this is currently precluded by the difficulty in sorting cells based on detection of HBZ protein in naturally-infected PBMCs. The role of the integration site in driving proliferation via either Tax or HBZ expression is also complicated by the effect of Tax and HBZ on the expression or function of each other.

A recent study in primary infection with BLV, a related retrovirus [27], has shown that early in infection, integration is favoured in transcriptionally active areas but is strongly selected against by the host immune response. Yet in subsequent chronic infection, abundant clones have a higher frequency of integration sites in transcriptionally active areas. Similarly, in HTLV-1, the effectiveness of the initial host response against expressed viral proteins is likely to define PVL set point, selecting against highly active clones. Clones in heterochomatin may also represent a dead end for the virus, because it may never be re-expressed. During lifelong chronic infection, however, surviving clones with integration sites in ‘intermediate’ transcriptionally active areas may have a proliferative advantage, although other factors (including clone TCR specificity or immune escape by Tax silencing or timing of viral expression [16, 23, 28]) will also contribute to the relative success of a clone. These transcriptionally active clones are more common in ACs than HAM/TSP, plausibly because they compete against the effective host response in ACs, or less likely, because they are selectively lost (by an unknown mechanism) in HAM/TSP.

There are other differences between HAM/TSP patients and AC individuals, in addition to the effectiveness of the T-cell immune response, which could alter the selection of proviral integration site during chronic infection. For example, in HAM/TSP, proliferation of HTLV-1-infected T cells may be maintained by IL-2 [29] and IL-15 [30], which may reduce the advantage conferred by integration sites that increase expression of proliferation-inducing factors such as HBZ.

Our results reflect systematic differences in the characteristics of HTLV-1 integration sites that persist in vivo between HAM/TSP patients and ACs. We propose that these differences are not themselves causative of the disparate clinical outcomes, but rather they reflect an underlying difference between patients with HAM/TSP and ACs in the efficiency of host-mediated control of HTLV-1 replication, for which there is extensive evidence [8].

Two previous studies compared the integration site environment between ACs and patients with HAM/TSP and found no differences, or a borderline significant (p = 0.049) difference [24, 26]. The difference between these reports and the present study may be attributable to the differences in sample size (N = 238 in present study, cp. N = 40 and 24 respectively in [24, 26]), the quantitative nature and greater sensitivity of the present high-throughput method, or in the ethnicity of the study population (uniformly southern Japanese vs. predominantly Caribbean). The incidence of HAM/TSP is much lower in the Japanese population (studied here) than in individuals of Caribbean origin in the previous studies (0.25 vs 3% [31, 32]). In the current study, we excluded differences in LM-PCR efficiency and mean clone abundance as causes of the observed differences between patients with HAM/TSP and ACs.

## Conclusions

The expression levels of Tax and HBZ influence both the rate of proliferation and the rate of CTL killing of each infected T-cell clone, and thus its equilibrium abundance in vivo. The balance between the relative strength of these opposing effects differ between the two genes: *tax* encodes the dominant target of the CTL response and is often silenced in abundant clones despite its capacity to support proliferation, but HBZ expression is typically maintained and is a poorer target for the CTL response. We have previously reported features of the integration site that influence Tax expression but there are no studies directly linking integration site and HBZ expression. We show that integration sites within genes and active genomic regions are more frequent in AC clones compared to equivalently-sized clones from HAM/TSP patients, and in individuals who have HLA class I allelesable to effectively present HBZ peptides to CTL. We postulate that integration in a transcriptionally active area may elevate HBZ and/or Tax expression and increase the equilibrium abundance of a clone. The increased frequency of integration sites observed in transcriptionally active genomic regions in ACs is consistent with the conclusion that greater proliferation is required to reach a given clonal abundance under the selection pressure exerted by an effective anti-HTLV-1 immune response.

## Methods

### Subjects

Kagoshima cohort. The study population has been previously reported [7, 10]. Subjects consisted of 229 patients with HAM/TSP and 202 HTLV-1-infected asymptomatic carriers randomly selected from blood donors; all were of Japanese ethnic origin and residing in Kagoshima, Kyushu, Japan [7, 10].

Kumamoto cohort. The study population consisted of 98 HTLV-1-infected asymptomatic carriers from blood donors in Kumamoto Prefecture, Kyushu, Japan.

Research was carried out in compliance with the Helsinki Declaration. The study was approved by the Faculty of Medical and Pharmaceutical Sciences Ethics Review Board, University Hospital, Kumamoto, Japan (Ethics 149). All patients gave written, informed consent for the study and for publication of anonymized results.

### HTLV-1 proviral load measurements

HTLV-1 DNA was amplified by quantitative PCR in a ABI7900HT FAST real time PCR system using FastSYBR Green (Applied Biosystems) reagents with the Tax-specific primers SK43 and SK44. A control region in B-actin was a**l**so amplified using ActF and ActR primers. The rat cell line TARL-2, which contains one integrated copy of the HTLV-1 provirus, was used to generate a standard curve. The sample copy number was interpolated from the standard curve and PVL was expressed as number of infected cells per 100 PBMCs. Proviral load data for the Kagoshima HAM/TSP and AC cohorts, with TARL-2 as pX region control, were as previously described ( [7]). SK43: 5′CGGATACCCAGTCTACGTGT, SK44: 5′GAGCCGATAACGCGTCCATCG, ActF: 5′TCACCCACACTGTGCCCATCTATGA, ActR: 5′ CATCGGAACCGCTACTTGCCGATAG.

### HLA class I alleles

HLA class I typing of the Kagoshima cohort was reported previously ( [10]). HLA typing of the Kumamoto cohort was done by Luminex reverse SSOP at the Hammersmith Hospital, London, UK to 2 digit resolution with ’strings’ of possible 4 digit resolution alleles. For each individual’s string of possible alleles, the most frequent 4-digit allele in the Japanese population (represented by a study of 1018 Japanese individuals [33]) was identified as the most likely allele. If there were multiple possible alleles with a population frequency >3%, all these frequent alleles were retained as possibilities for the individual. If no allele subtype in the string was represented in the population study, all alleles in the string were retained.

### Epitope binding prediction

We used the Metaserver algorithm (detailed in [21]) to predict HLA class I epitopes. Metaserver combines predicted TAP transport, proteasomal cleavage and HLA–peptide binding from NetCTL and NetMHC to predict peptide binding to HLA Class I A and B alleles present in the Kagoshima and Kumamoto cohorts. For each HLA class I A or B allele, all HTLV-1 peptides were ranked by binding score. The rank of the top HBZ peptide was recorded for each HLA class I allele. The rank of top binding peptide for a protein is a more robust method for comparisons between alleles than affinity [21]. HLA class alleles where the rank of the top HBZ peptide was < 20 were included as predicted binders of HBZ. A0201 was also included as a binder (HBZ top rank = 29) as it had been experimentally shown to present HBZ peptides [34]. For alleles not included in Metaserver, we assessed the rank of the binding score using NetMHC only. Alleles where the rank of the top HBZ peptide was < 20 were added to the predicted binders of HBZ. Approximately 30% of individuals have a detectable CTL response to HBZ [35]. Individuals with two or more HBZ-binding alleles constituted 39% of the combined cohorts, and were designated ‘strong HBZ binders’ representing individuals who were most likely to be able to mount an HBZ-specific response.

### High-throughput linker-mediated PCR identification of proviral integration sites

Integration site analysis was carried out on samples from patients with HAM/TSP and ACs from the Kagoshima cohort where sufficient genomic DNA was available (HAM/TSP, n = 104; AC, n = 100). To maximise statistical power and to test for reproducibility, we also analysed DNA of ACs from a neighbouring prefecture in Japan, Kumamoto (n = 98).

HTLV-1 integration sites were mapped and their abundance quantified as previously described ( [23, 24]). Genomic DNA from peripheral blood mononuclear cells was randomly sheared by sonication and ligated to a partially double-stranded DNA adaptor that incorporated a 6 nt barcode, a reverse sequencing primer site and the P7 sequence for paired-end sequencing. Two rounds of nested PCR were performed between the HTLV-1 LTR and the adaptor, adding a paired end P5 sequence in the LTR primer. The resulting amplicons were combined into libraries of up to 42 samples and sequence data were acquired on an Illumina GAII or HiSeq platform with paired-end 50 bp reads and a 6 bp index (barcode) read. Paired reads were aligned to a human genome reference (build 18, excluding haplotype and “random” chromosomes) using ELAND. A random set of integration sites was derived from ~ 190,000 50 bp human genome sequences generated using Galaxy, and aligned to the same human genome reference to control for any bias due to alignment limitations.

Unique integration sites (defined by Read1) were quantified on the basis of number of distinct shear sites identified (determined from paired Read2) and calibrated to provide a count of number of sequenced sister cells per clone. The absolute abundance (*Aabs*) of a T cell clonal population, defined by a single HTLV-1 integration site, was calculated as follows:


where *Si* is the number of sister cells of the *i*th clone, T is the total number of observed clones in the patient, and *PVL* is the number of infected cells per 100 PBMC in the patient. Clones were assigned to absolute abundance ranges of <1 per 10^4^ PBMC, 1-10 per 10^4^ PBMC, >10 per 10^4^ PBMC. LM-PCR integration site data sets for each patient were subjected to successive quality control filters as previously described ( [23, 24]); LM-PCR was designated ‘successful’ and the data included in further analysis only when the sample contained a minimum of 50 sister cells, a minimum of 15 clones, an average of more than 15 sequence reads per sister cell.

### Oligoclonality index

To measure the diversity of clone abundance in the infected cell population from each individual, we used the oligoclonality index [24] which is based on the Gini Index. This index measures the non-uniformity of the distribution of clone abundance: a value of 0 indicates that all clones have the same abundance and 1 is an upper bound where the proviral load effectively consists of a single clone.

### Estimating total number of clones in the blood

Estimation of total numbers of clones in the blood (observed and unobserved) was carried out using a computational diversity estimation approach (DivE), as described previously [25]. Briefly, many mathematical models were fitted to species-accumulation data, and to successively smaller nested subsamples thereof. Novel criteria were used to score models in how consistently they can reproduce existing observations from incomplete data. The estimates from the best performing models were aggregated (using the geometric mean) to estimate the number of clones in the circulation.

### Genetic and epigenetic analysis of integration site

Analysis of the genomic region surrounding the integration site was carried out as previously described ( [23, 24]). Specifically, the following attributes were identified for each integration site: location within/outside a transcriptional unit and orientation of the provirus versus that gene, proximity to a CpG island, and counts of selected histone marks within a 10 Kb window around the integration site. Locations of transcriptional units were retrieved from the NCBI (http://ftp.ncbi.nih.gov/gene/), CpG island data from UCSC tables [36], and histone marks from ChIP-seq experiments on primary CD4+ T cells (detailed in [24]). integration site positions were compared to the locations of specific relevant annotations using the R package hiAnnotator (http://malnirav.github.com/hiAnnotator), kindly provided by N. Malani and F. Bushman (University of Pennsylvania, USA). An integration site was designated as being in an area enriched in a particular histone mark if there were more counts of the mark within a window of 10 Kb around the integration site than around 90% of random sites.

### Statistical analysis

Statistical tests were performed using R version 2.15 (http://www.R-project.org/). A multivariable linear regression model was used to compare log PVL with the number of HBZ-binding HLA alleles per individual, taking into account disease status, as well as age and sex in the Kagoshima cohort-only analysis as these have been suggested to vary with PVL. Differences in OCI, PVL and total number of sisters between cohorts were analysed by non-parametric Mann-Whitney U tests as distributions were non-normal. Spearman correlation was used to determine the correlation in each cohort between log PVL and either log estimated number of clones in the blood or OCI. A likelihood ratio test was used to compare a null multivariable linear regression model associating log clone number with log PVL, disease status and HBZ binding capacity to an alternative model which added an interaction term between PVL and HBZ binding capacity. This allowed a test of whether the association between total clones in the blood and PVL differed with HBZ binding capacity. A second likelihood ratio test compared the same null model with a different alternative model including an interaction term between log PVL and disease status. This method was repeated for the association of OCI with log PVL.

In the genomic environment analysis, integration sites were grouped by absolute abundance range, and by the prefecture, disease status and HBZ binding status of the patient. For integration site data statistical analysis, the two AC cohorts were combined into a single AC group as they showed very similar integration site characteristics. Within subsets, the proportion of integration sites located within genes was plotted as odds ratio compared to random integration sites. Chi-squared tests were used to compare the total numbers of UIS lying inside or outside genes at each absolute abundance bin level. A chi-squared test for trend was used to measure the significance of a trend within a cohort across bins of increasing abundance. We plotted the mean number of a specified epigenetic mark within a 10 Kb window around integration sites within a group (N) divided by the mean number of that mark within 10 Kb of in silico random sites (N random). Mann-Whitney U tests were used to compare the numbers of specified epigenetic marks near integration sites from ACs versus HAM/TSP patients. Spearman correlation was used to test the association between log absolute abundance of an integration site clone and the frequency of a specified epigenetic mark within 10 Kb of the integration site.

Correction for multiple comparisons was made using a Bonferroni-Holm correction to control the family-wise error rate for each set of tests (Mann-Whitney, Spearman) carried out across all analysed epigenetic marks and integration site subsets. Two epigenetic marks (H3K9ac, H3K4ac) were analysed but not reported as their results were very similar to reported ones; the p values from their analyses were included in the calculation of the Bonferroni-Holm correction. Correction for multiple comparisons was also made across subsets in the analyses (Chi-square, Chi square test for trend) of integration sites in genes.

A multivariable logistic regression model was used to test whether disease status was independently associated with integration site in a gene, active genomic region and inhibitory genomic region at the integration site level (all integration sites used in analysis) or patient level (characteristics of integration sites averaged per patient). Host HBZ binding status and PVL, as well as log absolute clone abundance were controlled as additional factors in the model as these may vary with disease status and integration site environment. A multivariable logistic regression model was also used to test the association of integration in the same orientation as a gene or TSS with disease status (controlling for HBZ binding status, PVL and clone abundance). A further multivariable logistic regression tested the association of host HBZ binding status with integration in a gene (controlling for disease status and clone abundance) at the patient and integration site level.

## Electronic supplementary material

Additional file 1: Figure S1: Clone sister numbers, PVL and abundance classification in sequenced samples. **Figure S2.** Integration in a gene does not vary by viral load or AC cohort and there is no association of disease status of orientation with respect to flanking gene. **Figure S3.** Integration in transcriptionally active regions is more frequent in asymptomatic controls than HAM/TSP patients. **Figure S4.** Transcriptionally active integration sites are more frequent in AC than HAM/TSP patients. (PDF 137 KB)

## Abbreviations

AC: Asymptomatic carrier
ATLL: adult T cell leukaemia/lymphoma
CTL: Cytotoxic lymphocyte
HAM/TSP: HTLV-1-associated myelopathy/tropical spastic paraparesis
HBZ: HTLV-1 basic leucine zipper factor
HTLV-1: Human T cell lymphotropic virus-type 1
OCI: Oligoclonality index
LM-PCR: Linker mediated polymerase chain reaction
PBMC: Peripheral blood mononuclear cell
PVL: Proviral load.

## References

[1] Gessain A, Cassar O (2012). Epidemiological Aspects and World Distribution of HTLV-1 Infection. Front Microbiol.

[2] Cook LB, Rowan AG, Melamed A, Taylor GP, Bangham CRM (2012). HTLV-1-infected T cells contain a single integrated provirus in natural infection. Blood.

[3] Arnold J, Zimmerman B, Li M, Lairmore MD, Green PL (2008). Human T-cell leukemia virus type-1 antisense-encoded gene, Hbz, promotes T-lymphocyte proliferation. Blood.

[4] Satou Y, Yasunaga J-i, Yoshida M, Matsuoka M (2006). HTLV-I basic leucine zipper factor gene mRNA supports proliferation of adult T cell leukemia cells. Proc Natl Acad Sci.

[5] Boxus M, Willems L (2009). Mechanisms of HTLV-1 persistence and transformation. Br J Cancer.

[6] Yamano Y, Sato T (2012). Clinical pathophysiology of human T-lymphotropic virus-type 1-associated myelopathy/tropical spastic paraparesis. Front Microbiol.

[7] Nagai M, Usuku K, Matsumoto W, Kodama D, Takenouchi N (1998). Analysis of HTLV-I proviral load in 202 HAM/TSP patients and 243 asymptomatic HTLV-I carriers: high proviral load strongly predisposes to HAM/TSP. J Neurovirol.

[8] Bangham CRM (2009). CTL quality and the control of human retroviral infections. Eur J Immunol.

[9] Journo C, Mahieux R (2011). HTLV-1 and innate immunity. Viruses.

[10] Jeffery KJ, Usuku K, Hall SE, Matsumoto W, Taylor GP, Procter J, Bunce M, Ogg GS, Welsh KI, Weber JN, Lloyd AL, Nowak MA, Nagai M, Kodama D, Izumo S, Osame M, Bangham CR (1999). HLA alleles determine human T-lymphotropic virus-I (HTLV-I) proviral load and the risk of HTLV-I-associated myelopathy. Proc Natl Acad Sci U S A.

[11] Goon PKC, Biancardi A, Fast N, Igakura T, Hanon E, Mosley AJ, Asquith B, Gould KG, Marshall S, Taylor GP, Bangham CRM (2004). Human T cell lymphotropic virus (HTLV) type-1-specific CD8+ T cells: frequency and immunodominance hierarchy. J Infect Diseases.

[12] Kannagi M, Harada S, Maruyama I, Inoko H, Igarashi H, Kuwashima G, Sato S, Morita M, Kidokoro M, Sugimoto M (1991). Predominant recognition of human T cell leukemia virus type I (HTLV-I) pX gene products by human CD8+ cytotoxic T cells directed against HTLV-I-infected cells. Int Immunol.

[13] Niewiesk S, Daenke S, Parker CE, Taylor G, Weber J, Nightingale S, Bangham CR (1995). Naturally occurring variants of human T-cell leukemia virus type I Tax protein impair its recognition by cytotoxic T lymphocytes and the transactivation function of Tax. J Virol.

[14] Koiwa T, Hamano-usami A, Ishida T, Okayama A, Yamaguchi K, Kamihira S, Koiwa T, Hamano-usami A, Ishida T, Okayama A, Yamaguchi K, Kamihira S, Watanabe T (2002). 5′-Long Terminal Repeat-Selective CpG Methylation of Latent Human T-Cell Leukemia Virus Type 1 Provirus In Vitro and In Vivo. J Virol.

[15] Tamiya S, Matsuoka M, Etoh K, Watanabe T, Kamihira S, Yamaguchi K, Takatsuki K (1996). Two types of defective human T-lymphotropic virus type I provirus in adult T-cell leukemia. Blood.

[16] Cook LB, Melamed A, Niederer H, Valganon M, Laydon D, Foroni L, Taylor GP, Matsuoka M, Bangham CRM (2014). The role of HTLV-1 clonality, proviral structure and genomic integration site in adult T cell leukemia/lymphoma. Blood.

[17] Furukawa Y, Kubota R, Tara M, Izumo S, Osame M (2001). Existence of escape mutant in HTLV-I tax during the development of adult T-cell leukemia. Blood.

[18] Asquith B, Mosley AJ, Barfield A, Marshall SEF, Heaps A, Goon P, Hanon E, Tanaka Y, Taylor GP, Bangham CRM (2005). A functional CD8+ cell assay reveals individual variation in CD8+ cell antiviral efficacy and explains differences in human T-lymphotropic virus type 1 proviral load. The Journal of General Virology.

[19] Sabouri AH, Usuku K, Hayashi D, Izumo S, Ohara Y, Osame M, Saito M (2008). Impaired function of human T-lymphotropic virus type 1 (HTLV-1)-specific CD8+ T cells in HTLV-1-associated neurologic disease. Blood.

[20] Saito M, Matsuzaki T, Satou Y, Yasunaga J-I, Saito K, Arimura K, Matsuoka M, Ohara Y (2009). In vivo expression of the HBZ gene of HTLV-1 correlates with proviral load, inflammatory markers and disease severity in HTLV-1 associated myelopathy/tropical spastic paraparesis (HAM/TSP). Retrovirology.

[21] Macnamara A, Rowan A, Hilburn S, Kadolsky U, Fujiwara H, Suemori K, Yasukawa M, Taylor G, Bangham CRM, Asquith B (2010). HLA class I binding of HBZ determines outcome in HTLV-1 infection. PLoS Pathog.

[22] Van Dooren S, Pybus OG, Salemi M, Liu H-F, Goubau P, Remondegui C, Talarmin A, Gotuzzo E, Alcantara LCJ, Galvão-Castro B, Vandamme A-M (2004). The low evolutionary rate of human T-cell lymphotropic virus type-1 confirmed by analysis of vertical transmission chains. Mol Biol Evol.

[23] Melamed A, Laydon DJ, Gillet NA, Tanaka Y, Taylor GP, Bangham CRM (2013). Genome-wide determinants of proviral targeting, clonal abundance and expression in natural HTLV-1 infection. PLoS Pathog.

[24] Gillet NA, Malani N, Melamed A, Gormley N, Carter R, Bentley D, Berry C, Bushman FD, Taylor GP, Bangham CRM (2011). The host genomic environment of the provirus determines the abundance of HTLV-1-infected T cell clones. Blood.

[25] Laydon DJ, Melamed A, Sim A, Gillet NA, Sim K, Darko S, Kroll JS, Douek DC, Price DA, Bangham CRM, Asquith B (2014). Quantification of HTLV-1 clonality and TCR diversity. PLoS Comput Biol.

[26] Meekings KN, Leipzig J, Bushman FD, Taylor GP, Bangham CRM (2008). HTLV-1 integration into transcriptionally active genomic regions is associated with proviral expression and with HAM/TSP. PLoS Pathog.

[27] Gillet NA, Gutiérrez G, Rodriguez SM, de Brogniez A, Renotte N, Alvarez I, Trono K, Willems L (2013). Massive depletion of bovine leukemia virus proviral clones located in genomic transcriptionally active sites during primary infection. PLoS Pathog.

[28] Gillet NA, Cook L, Laydon DJ, Hlela C, Verdonck K, Alvarez C, Gotuzzo E, Clark D, Farre L, Bittencourt A, Asquith B, Taylor GP, Bangham CRM (2013). Strongyloidiasis and infective dermatitis alter human T lymphotropic virus-1 clonality in vivo. PLoS Pathog.

[29] Tendler CL, Greenberg SJ, Blattner W, Manns A, Murphy E, Fleisher T, Hanchard B, Morgan O, Burton JD, Nelson DL (1990). Transactivation of interleukin 2 and its receptor induces immune activation in human T-cell lymphotropic virus type I-associated myelopathy: pathogenic implications and a rationale for immunotherapy. Proc Natl Acad Sci U S A.

[30] Azimi N, Mariner J, Jacobson S, Waldmann TA (2000). How does interleukin 15 contribute to the pathogenesis of HTLV type 1-associated myelopathy/tropical spastic paraparesis?. AIDS Res Hum Retroviruses.

[31] Kaplan JEOM, Kubota H, Igata A, Nishitani H, Maeda Y, Khabbaz RF, Janssen RS (1990). The risk of development of HTLV-I-associated myelopathy/tropical spastic paraparesis among persons infected with HTLV-I. J Acquir Immune Defic Syndr.

[32] Tosswill JHC, Taylor GP, Tedder RS, Mortimer PP, Voss LD, Mulligan J (2000). HTLV-I/II associated disease in England and Wales, 1993–7: retrospective review of serology requests. Br Med J.

[33] Itoh Y, Mizuki N, Shimada T, Azuma F, Itakura M, Kashiwase K, Kikkawa E, Kulski JK, Satake M, Inoko H (2005). High-throughput DNA typing of HLA-A, -B, -C, and -DRB1 loci by a PCR-SSOP-Luminex method in the Japanese population. Immunogenetics.

[34] Suemori K, Fujiwara H, Ochi T, Ogawa T, Matsuoka M, Matsumoto T, Mesnard J-M, Yasukawa M (2009). HBZ is an immunogenic protein, but not a target antigen for human T-cell leukemia virus type 1-specific cytotoxic T lymphocytes. J Gen Virol.

[35] Hilburn S, Rowan A, Demontis M-A, MacNamara A, Asquith B, Bangham CRM, Taylor GP (2011). In vivo expression of human T-lymphotropic virus type 1 basic leucine-zipper protein generates specific CD8+ and CD4+ T-lymphocyte responses that correlate with clinical outcome. J Infect Dis.

[36] Karolchik D, Hinrichs AS, Furey TS, Roskin KM, Sugnet CW, Haussler D, Kent WJ (2004). The UCSC Table Browser data retrieval tool. Nucleic Acids Res.

