# Supplementary material for: HTLV-1 proviral integration sites differ between asymptomatic carriers and patients with HAM/TSP
Source: Virol J. 2014 Sep 30;11:172. doi: 10.1186/1743-422X-11-172 (PMC4192323; doi:10.1186/1743-422X-11-172)
Supplement: Supplementary file 1 — Additional file 1: Figure S1: Clone sister numbers, PVL and abundance classification in sequenced samples. Figure S2. Integration in a gene does not vary by viral load or AC cohort and there is no association of disease status of orientation with respect to flanking gene. Figure S3. Integration in transcriptionally active regions is more frequent in asymptomatic controls than HAM/TSP patients. Figure S4. Transcriptionally active integration sites are more frequent in AC than HAM/TSP patients. (PDF 137 KB) [file 12985_2014_2499_MOESM1_ESM.pdf]

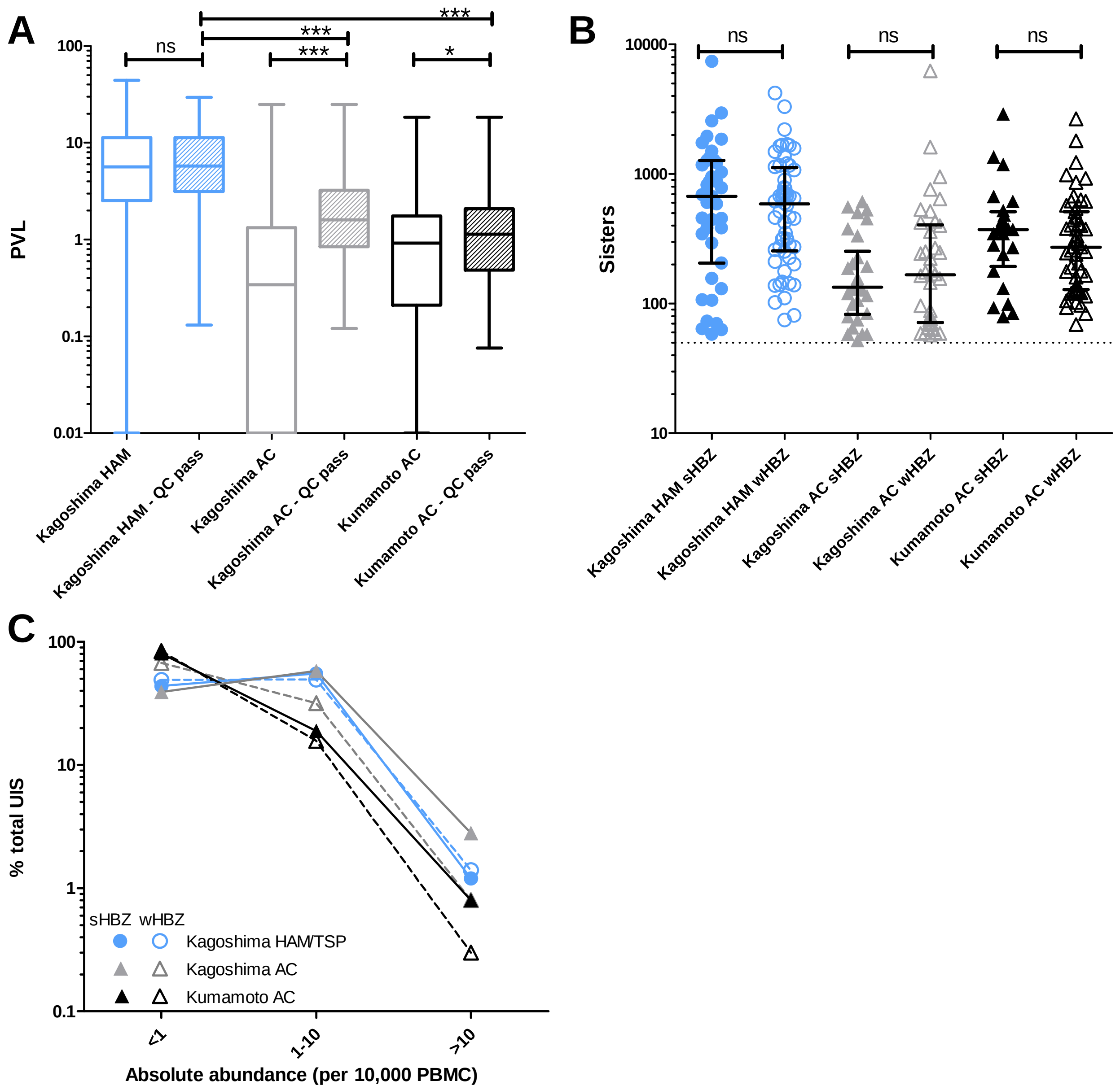

Additional Figure 1: Clone sister numbers, PVL and abundance classification in sequenced samples

HTLV-1 unique integration sites (UIS) from Japanese asymptomatic carriers (AC, triangles) from Kagoshima (grey) and Kumamoto (black) were compared to those from HAM/TSP patients (HAM/TSP, blue, circles, from Kagoshima). Genomic DNA samples were processed by sonication-based LM-PCR. Samples passed quality checks (QC pass) if >15 integration site clones and >50 total clone 'sister' cells were detected. (A) There was no significant difference in proviral load (PVL) between the complete HAM/TSP cohort and those samples which were processed by LM-PCR and passed quality checks. AC cohorts showed an increase in median PVL in samples that passed QC checks as AC cohorts contained individuals with very low PVL which did not yield sufficient data. (B) UIS were stratified by predicted HBZ peptide binding affinity of host HLA class I alleles (strong binders, sHBZ, filled symbols; weak binders, wHBZ, open symbols).There was no significant difference in the median (line) number of sisters sequenced in LM-PCR samples which passed quality checks (dotted line: 50 sisters) between strong and weak HBZ binders. HAM/TSP had higher median sisters sequenced than AC, corresponding to a higher overall median PVL. (C) UIS clones were allocated to bins based on clone absolute abundance per 10,000 PBMC. In each cohort, there was a non-significant increase in the percentage of greater abundance clones in individuals who could bind HBZ epitopes. Statistical comparisons: Mann-Whitney U test; Significance: * 0.01<p<0.05, ** 0.001<p<0.01, *** p<0.001.

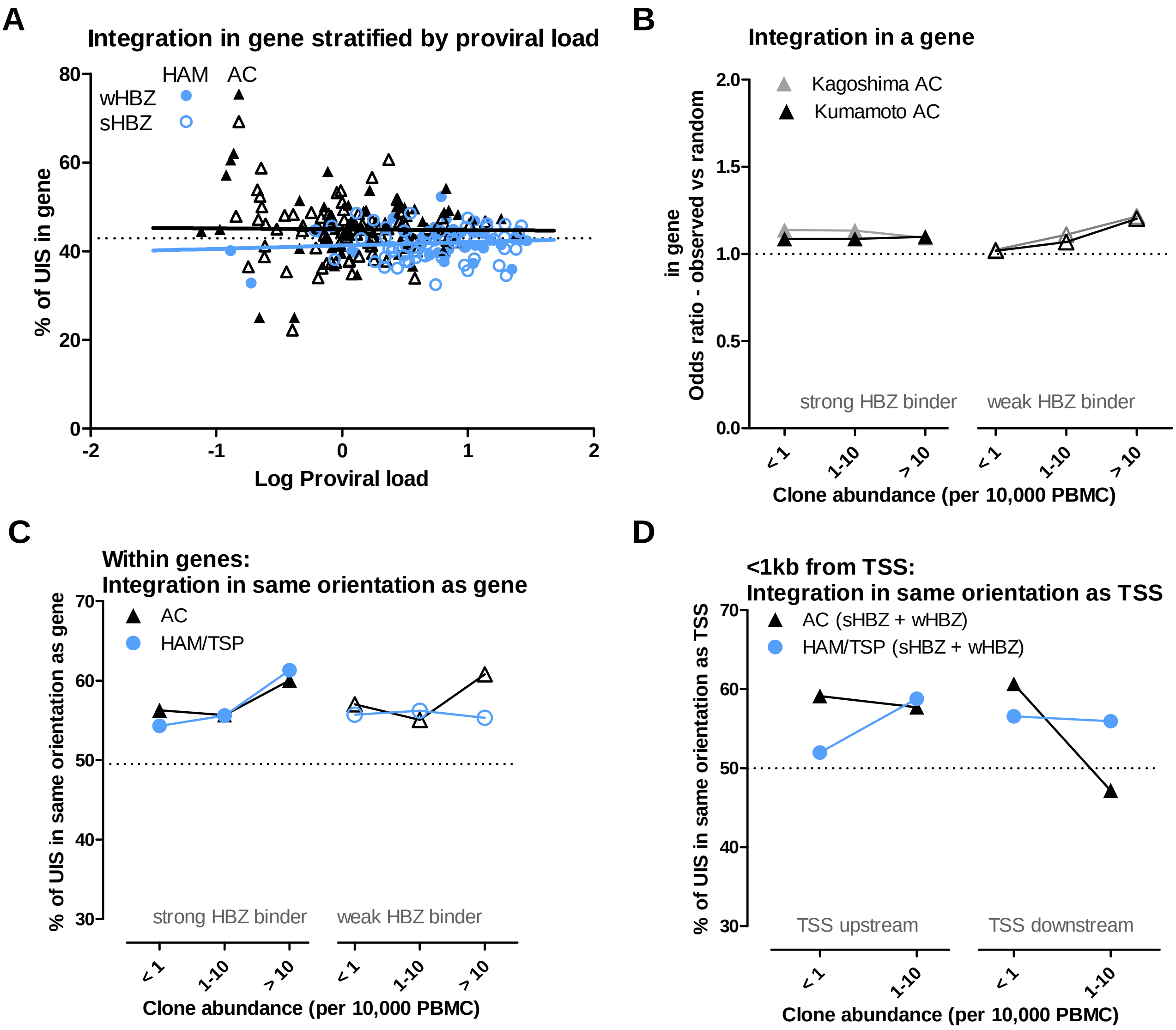

Additional Figure 2: Integration in a gene does not vary by viral load or AC cohort and there is no association of disease status of orientation with respect to flanking gene.

HTLV-1 unique integration sites (UIS) from Japanese asymptomatic carriers (AC, triangles) from Kagoshima and Kumamoto were compared to those from HAM/TSP patients (HAM/TSP, blue circles, from Kagoshima). UIS were stratified on the basis of predicted HBZ peptide binding affinity of host HLA class I alleles (strong binders, filled symbols; weak binders, open symbols) and integration site clone absolute abundance bin. An in silico generated random integration site dataset (dotted line) is used as a comparison. (A)Percent of UIS per sample which are located within a gene is not correlated with proviral load in any cohort (Spearman correlation). (B) There is no significant difference between percentage UIS within a gene in asymptomatic cohorts from Kagoshima (grey) or Kumamoto (black) (chi square test). (C) Integration sites within a gene are more likely to have the provirus in the same orientation as the gene; but neither disease status (p = 0.17, logistic regression) nor clone abundance consistently alters this preference. (D) Integration sites in the same orientation as a nearby (within 1kb) downstream or upstream transcriptional start site (TSS) are not consistently favoured in ACs compared to HAM/TSP (upstream p = 0.26, downstream p = 0.79; logistic regression).

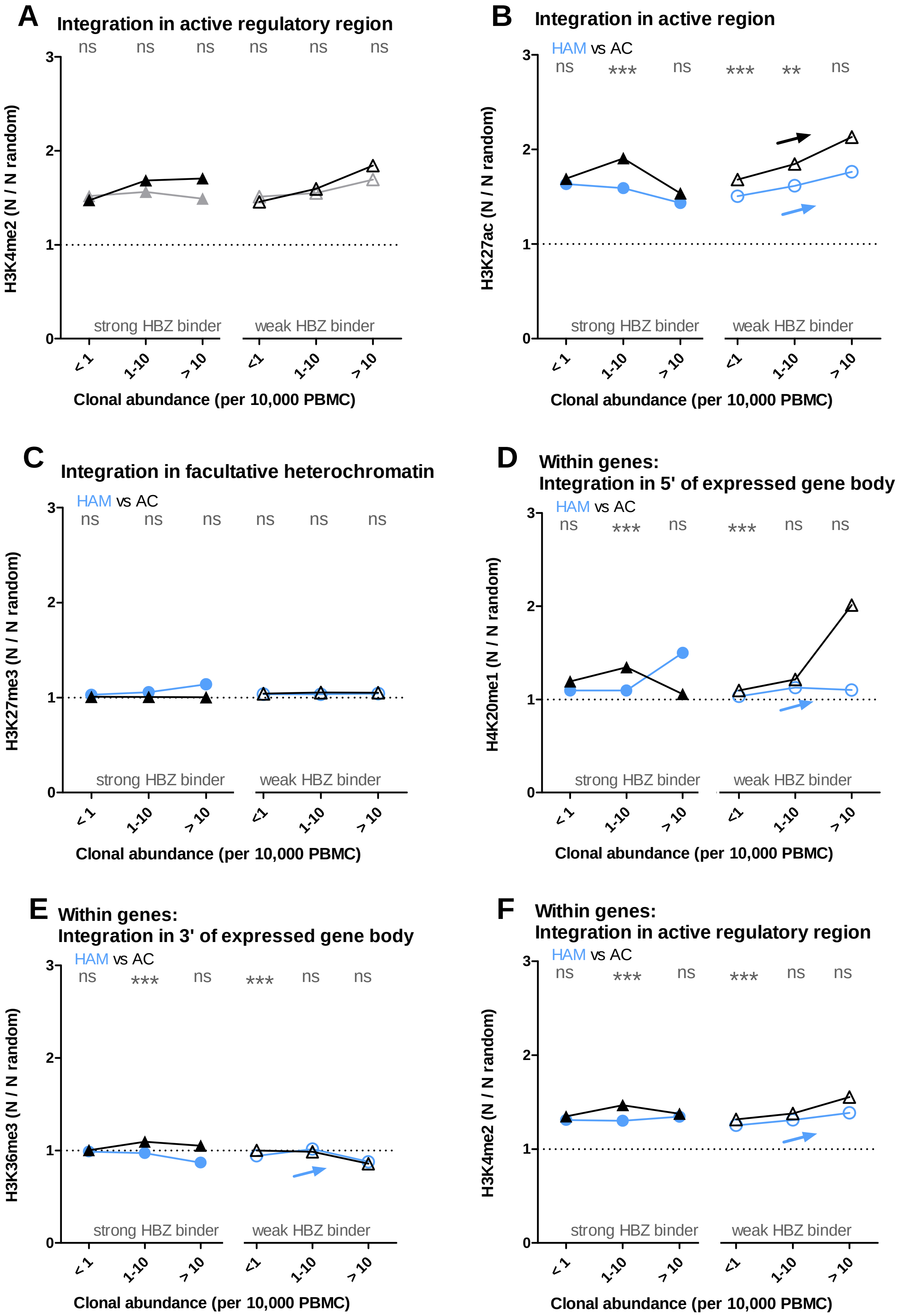

Additional Figure 3: Integration in transcriptionally active regions is more frequent in asymptomatic controls than HAM/TSP patients.

HTLV-1 integration sites from Japanese asymptomatic carriers from Kagoshima and Kumamoto prefectures (AC, triangles) were compared to those from HAM/TSP patients (HAM/TSP, blue circles, from Kagoshima).

Integration sites were stratified on the basis of predicted HBZ peptide binding affinity of host HLA class I alleles (strong binders, filled symbols; weak binders, open symbols) and integration site clone absolute abundance. Grouped integration sites are represented by a single symbol showing mean epigenetic mark frequency within 10Kb of integration sites divided by frequency near random sites. (A) There was no significant difference between AC from Kagoshima (grey) and Kumamoto (black) in any clone abundance group (Mann-Whitney U test). (B) Combined AC (black triangles) integration sites had a greater frequency of nearby H3K27ac marks (associated with active regulatory regions) than HAM/TSP patients. (C) No consistent association with frequency of H3K27me3 marks (enriched in facultative heterochromatin) was observed. (D) Proviruses integrated within genes had more higher numbers of marks associated with the 5' ends of active transcribed genes (H4K20me1) in ACs versus HAM/TSP patients. (E) There was no consistent association with H3K36me3, associated with the 3' end of expressed gene bodies. (F) Even for integration sites within genes, H3K4me2 marks were more frequent in AC compared to HAM/TSP, indicating association with an active region is distinct from that of integration in a gene. Statistical comparisons AC vs HAM: significant Mann-Whitney U Test corrected for multiple comparisons * $0.05>p>0.01$, ** $0.01>p>0.001$, *** $p<0.001$. Arrow: significant Spearman correlation between frequency of epigenetic marks and clone absolute abundance within a cohort.

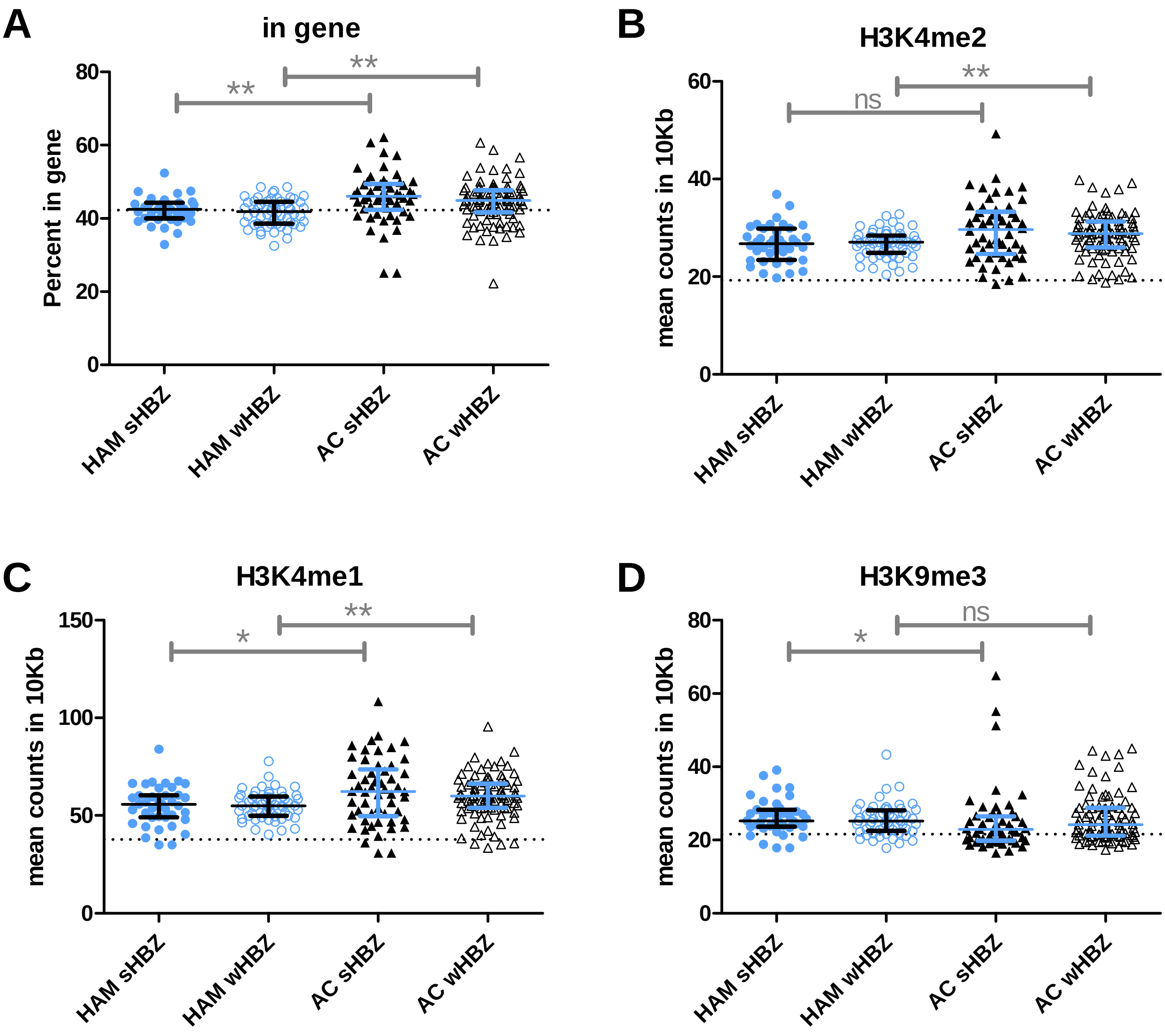

Additional Figure 4: Transcriptionally active integration sites are more frequent in AC than HAM/TSP patients.

HTLV-1 unique integration sites from Japanese asymptomatic carriers (AC, black triangles) from Kagoshima and Kumamoto were compared to those from HAM/TSP patients (blue circles, from Kagoshima). Patients were stratified on the basis of predicted HBZ peptide binding affinity of host HLA class I alleles (strong binders, sHBZ, filled symbols; weak binders, wHBZ, open symbols). Dotted line: in silico random sites. (A) AC individuals had a greater percentage of integration sites in genes than HAM/TSP patients. (B) Asymptomatic carriers had a higher frequency of H3K4me2 marks, enriched in transcriptionally active areas, within 10Kb of integration sites, and (C) a higher frequency than HAM/TSP of H3K4me1 marks, associated with enhancers. (D) In contrast, AC had a lower frequency of H3K9me3 marks, associated with constitutively heterochromatic DNA. There was no significant difference between sHBZ and wHBZ subsets within cohorts. Bars denote median +/- interquartile range. Statistical comparisons AC vs HAM by Mann-Whitney U test after correction for multiple testing: * 0.05>p>0.01, ** 0.01>p>0.001, *** p<0.001.
